# Artificial Gastrointestinal Models for Nutraceuticals Research—Achievements and Challenges: A Practical Review

**DOI:** 10.3390/nu14132560

**Published:** 2022-06-21

**Authors:** Anna Gościniak, Piotr Eder, Jarosław Walkowiak, Judyta Cielecka-Piontek

**Affiliations:** 1 Department of Pharmacognosy, Poznan University of Medical Sciences, Rokietnicka 3, 60-806 Poznan, Poland; anna.gosciniak@student.ump.edu.pl; 2 Department of Gastroenterology, Dietetics and Internal Diseases, Poznan University of Medical Sciences, Przybyszewskiego 49, 60-355 Poznan, Poland; piotreder@ump.edu.pl; 3 Department of Pediatric Gastroenterology and Metabolic Diseases, Poznan University of Medical Sciences, Szpitalna 27/33, 60-572 Poznan, Poland; jarwalk@ump.edu.pl

**Keywords:** microbiome, microbiota, in vitro models, human gut, nutraceuticals

## Abstract

Imitating the human digestive system as closely as possible is the goal of modern science. The main reason is to find an alternative to expensive, risky and time-consuming clinical trials. Of particular interest are models that simulate the gut microbiome. This paper aims to characterize the human gut microbiome, highlight the importance of its contribution to disease, and present in vitro models that allow studying the microbiome outside the human body but under near-natural conditions. A review of studies using models SHIME, SIMGI, TIM-2, ECSIM, EnteroMix, and PolyfermS will provide an overview of the options available and the choice of a model that suits the researcher’s expectations with advantages and disadvantages.

## 1. Introduction

The growing interest in the impact of the microbiome on human health calls for the development of studies that can it illustrate. Recent research suggests that disruption of the microbiome may impact several conditions such as diabetes and neurological disorders. The gut-brain axis is an interesting topic gaining recognition. The solution may be to modify the microbiome by nutrients or plant-based substances. However, the observation of relationships in vivo is hampered by difficult direct access to sections of the gastrointestinal tract, as well as by ethical concerns. Animal studies are also not adequate due to differences in anatomy, physiology and phylogenetic and coevolutionary differences between microbiota species in animal and human models. A solution may be to use in vitro models simulating gastrointestinal conditions. The models proposed by the researchers can simulate both stomach, small and large intestine conditions or only the large intestine. These models use faecal samples from human volunteers so that the microbiome’s composition is a good representation of reality. However, it is also possible to use single cultures to assess the effect on specific strains. The many opportunities to use in vitro models have encouraged the development of these methods, although they are not without their disadvantages.

## 2. Gut Microbiota

Almost 1000 different species of bacteria colonize the human gastrointestinal tract. Its surface is estimated at 250–400 m^2^, making it the second-largest system in terms of the surface area of the human body (the respiratory system has a greater surface area) [1]. The number and diversity of bacteria are relatively small in the stomach. This is determined by the extreme conditions of low pH, the presence of gastric juice, and the quick rate of content flow. The concentration of bacteria increases with the distance traveled by the chyme, from 10^3^ cells/mL in the duodenum and jejunum, 10^8^ cells/mL in the ileum to 10^11^–10^12^ cells/mL in the colon [2]. The term “microbiota” is usually defined as an assemblage of living microorganisms (not only bacteria, but also fungi, achreons, and others) found in a specific environment. The microbiome, on the other hand, refers to the entire environment, including microorganisms, their genomes, and surrounding environmental conditions [3]. In this publication, the microbiome and microbiota are mainly understood in terms of bacteria in the colon and the terms are used interchangeably with the understanding that the difference is nonsignificant in this context. Studies have shown that most of the gut microbiota consists of absolutely anaerobic microorganisms, followed by relatively anaerobic and aerobic microorganisms. Of these bacteria, most belong to the Firmicutes, Bacterioidetes, and Actinobacteria types. Proteobacteria, Fusobacteria, Cyanobacteria, and Verrucomicrobia are present in smaller amounts [4].

The intestinal microbiota changes during the human lifetime depending on age, physiological condition, diet, host immune mechanisms, drugs used, and other environmental factors. 

The dominant bacteria in the digestive tract change over the course of a person’s life. After the baby is born, the gastrointestinal tract of the newborn is populated by particular species of microorganisms. Even the first days of life and how infants are fed influence the formation of the gut microbio. Available data suggest that the mode of birth (natural delivery or cesarean section) is also crucial in the first stages of life. However, recent reports indicate that it is a much more sophisticated correlation [5]. Regardless of the mode of delivery, the microbiota is influenced by the mother’s organisms and the environment. Geographical location may also indirectly affect the microbiota in early life, but this is mainly due to a geographical location determining eating habits and lifestyle. At three years of age, the composition of a child’s microbiota begins to resemble that of adults. It has been noted that the composition of the gut microbiota varies at different periods of life, i.e., during adolescence, pregnancy, and menopause. It is supposed that sex hormones are responsible for changes in the gut microbiota [6].

### 2.1. Functions of the Gut Microbiome

Recent years have seen the increased interest of researchers in the subject of gut microbiota and its impact on the functioning of host organisms. Results have confirmed that the composition of intestinal flora is crucial for human health [7]. The main functions of bacteria colonizing the gut are the prevention of colonization by pathogens, synthesizingand modulation of the immunological system of the host [8,9]. Knowledge of the benefits of maintaining proper intestinal flora has triggered increased interest in health-promoting nutritional preparations that contain beneficial bacteria. Lifestyle, diet, and exposure to stress are known to cause disorders in the composition of the gut microbiome [10]. 

Research on intestinal microbiota is most advanced in humans. Significant progress has been observed in the field. The influence of gut microbiome composition on autoimmune diseases, colon cancer, tooth decay, and various nervous disorders, such as depression and autism, has been studied [11]. Moreover, intestinal bacteria participate in the maturation and exchange of enterocytes, immunomodulation, gastrointestinal tract motility, drug metabolism, breakdown of dietary toxins and carcinogens (e.g., heterocyclic amines, N-nitroso compounds), fermentation of undigested food ingredients and in the production of essential vitamins (K, B_12_, folic acid, B_1_, B_6_), in bile acid recirculation (by the production of bile acid hydrolases), and also in protection against intestine colonization by pathogenic bacteria, such as *Escherichia coli*, *Vibrio cholerae*, *Clostridium* spp., *Salmonella* spp., and *Shigella* spp. [12].

### 2.2. Short-Chain Fatty Acids (SCFAs)

Short-chain fatty acids (SCFAs) are produced by fermenting indigestible saccharides by the gut microbiome. The main products are acetate, propionate, and butyrate. SCFAs are involved in many functions, and disruption of their production is proposed as a mechanism linking the microbiome and, e.g., neurological descents. They are the primary energy source for colonocytes—butyrate is the main and preferred metabolic substrate. It provides at least 60–70% of the energy requirements necessary for their proliferation and differentiation [13]. Regulate epithelial barrier integrity—increased permeability is associated with bacteria translocation and cell wall components that activate the inflammatory cascade. 

Furthermore, both butyrate and propionate reportedly inhibit histone deacetylases (HDAC) [14]. HDAC play a key role in the homeostasis of protein acetylation in histones and the regulation of fundamental cellular activities such as transcription. HDAC inhibitors have neuroprotective and anti-inflammatory properties. As demonstrated in models, they improve neurological performance, learning and memory, and other disease phenotypes [15]. SCFAs also have a role in regulating energy balance. SCFAs bind to G protein-coupled receptors such as GPR43 and GPR41. This results in stimulated secretion of glucagon-like peptide 1 (GLP-1) and peptide YY (PYY) enhanced insulin secretion [16]. However, research on this topic is still developing, and no clear conclusions have yet been reached.

## 3. The Link between the Microbiome and Diseases

The link between a disturbance of the microbiome and diseases such as obesity, diabetes and neurological disorders has been the subject of research in recent years. The development of interests in these areas encourages further exploration of the topic using, among others, artificial models of the gastrointestinal tract. The following information provides some insight into the link between the microbiome and common conditions such as obesity and diabetes, as well as the impact on the nervous system. 

### 3.1. Obesity

Recently, the link between gut microbiome disorders and obesity has received increasing attention. One of the first studies to address this issue used a germ-free mouse model, i.e., mice lacking their own intestinal microbiome transplanted artificially. Turnbaugh et al. [17] demonstrated that the composition of the intestinal microbiome influences body mass. The authors transferred microorganisms obtained from the intestines of homozygotic obese leptin-deficient mice ob/ob and mice with proper body mass to “germ-free” mice. After two weeks, the mice treated with microorganisms obtained from the obese specimens took more calories from the feed and accumulated more significant amounts of fat tissue. Moreover, alteration of the intestinal microbiome was found to trigger inflammatory changes and obesity that result from the effects this alteration exerts on the epithelial and endocrine cells [18,19]. In addition, intestinal microbiome changes induce inflammation and obesity by affecting intestinal epithelial cells and enteroendocrine cells as well as the secretion of intestinal hormones: glucagon-like peptides 1 and 2 (GLP-1 and GLP-2). GLP-1 stimulates insulin secretion, delays the passage of food through the stomach, induces satiety and weight loss, GLP-2 increases glucose transport from the intestines and reduces the permeability of the intestinal wall. Thus, the microbiome affects metabolism, acting on enteroendocrine cells [20]. On the other hand, it is still debated whether gut microbiome disorders are a cause of obesity or an effect of an unhealthy diet. 

### 3.2. Diabetes

Several mechanisms linking type 2 diabetes (T2D) and the gut microbiome have been proposed. One of these is increased intestinal permeability leading to metabolic endotoxemia, a low-grade inflammatory response, and an immune response triggered by Toll-like receptor binding. This leads to the development of insulin resistance [21]. Too much LPS can destroy the integrity of the intestinal barrier and increase LPS absorption. A way to prevent this may be to provide SCFAs that benefit the integrity of the intestinal barrier—mainly butyrate. SCFAs also have a beneficial effect on glucose metabolism by activating L-cell G-proteins to promote the release of GLP-1 and peptide YY (PYY) [22]. Probiotic strains that were effective in modulating glucose levels were tested for diabetes prevention. *Lactobacillus reuteri* fed to high fructose-fed rats reduced T2D markers such as serum glucose, glycated hemoglobin, and c-peptide. Palacios et al. [23] showed that the combination of metformin with multi-strain probiotic (*L. plantarum*, *L. bulgaricus, L. gasseri, B. breve*, *B. animalis* sbsp. *lactis*, *B. bifidum*, *S. thermophilus*, and *S. boulardii*) leads to improvements in fasting plasma glucose, insulin resistance, and the permeability marker zonulin, with beneficial changes in SCFA-producing bacteria. However, no significant changes in metabolic, inflammatory, and permeability markers were observed between the probiotic and placebo groups. An interesting strain is *A. muciniphila* which is thought to reduce insulin resistance and decrease the destruction of the intestinal barrier. *A. muciniphila* is less abundant in pre-diabetic patients and also among newly diagnosed T2D patients. *A. muciniphila* can reduce low-grade inflammatory reactions and metabolic disorders [24]. In a study by Depommiera et al. [25] compared with placebo, pasteurised *A. muciniphila* improved insulin sensitivity and reduced insulinaemia and total plasma cholesterol.

### 3.3. Nervous System

One of the interesting issues concerning the human microbiome is the impact of the microbiota on the nervous system and neurodegenerative diseases. One of the theories connecting gut microbiome and neurodegenerative disease links gut bacteria to immune activation through a defective gut barrier. This pathogenic permeability results in a systemic inflammatory response that impairs the blood–brain barrier and promotes neuroinflammation and eventually neuronal damage and degeneration [26]. It is suggested that microbiome is a “second brain” and pathway of communication is named gut-brain-axis is responsible for some neurodegenerative disorders such as Alzheimer’s disease Parkinson. Some bacterial strains can modify the levels of neurotransmitter precursors in the intestinal lumen and even independently synthesize or modulate the synthesis of such as neurotransmitters, including γ-aminobutyric acid (GABA), serotonin (5-HT), dopamine (DA), and norepinephrine (NA) [14]. This, in turn, may influence the development of neurodegenerative diseases. However, the effects on neuronal function involve broader mechanisms. 

Since these diseases are primarily associated with the elderly, the accompanying problem is increased permeability of blood-brain-barrier and consequently facilitated enters of harmful elements such as bacterial LPS [27]. In a study conducted by Bonfili et al. [28] on a mouse model of Alzheimer’s disease, administration of lactic acid bacteria and bifidobacteria showed to change the composition of the gut microbiota and its metabolites, positively affecting inflammatory cytokines, gut hormone levels, and proteolysis, reducing Aβ load and improving cognitive function. Butyrate administration restored memory function and increased the expression of genes involved in associative learning in a mouse model, which was associated with HDAC inhibition by SCFAs [29].

## 4. Models of the Human Gastrointestinal Tract In Vitro

In recent years, several models have been developed that can simulate gastrointestinal conditions and can be used to assess the effects of active substances on the gastrointestinal tract. A review of the application of the models shows that a particularly interesting topic is the effect of substances of natural origin, especially polyphenols, on the human microbiome. The use of in vitro models for this purpose is highly desirable. However, a major problem with studies of effects on the human microbiome is the lack of general guidelines. Each of the models presented has different conditions concerning, for example, pH at different sections of the intestine, fluid volumes used, or composition of the medium. This lack of systematization leads to discrepancies in the way the tests are carried out and in the results obtained. Awareness of the differences between models their advantages and disadvantages will allow the researcher to use these methods with full knowledge. However, the consensus in carrying out these methods would be highly desirable.

### 4.1. SHIME Simulator of the Human Intestinal Microbial Ecosystem 

SHIME is a dynamic in vitro simulator model of the human digestion system developed in 1993 and successfully used today [30]. SHIME simulates upper gastrointestinal conditions with a total of five compartments simulating the upper (stomach, small intestine) and lower (ascending, transverse and descending colon) gastrointestinal tract [31]. The reactor contains five glass vessels in double shells at 37 °C, which are connected by peristaltic pumps. Testing time may vary from 24 to 76 h. Because of the necessity to maintain anaerobic conditions in the lower gastrointestinal tract, daily rinsing of the space above the contents of the respective chambers with N_2_ gas or 90/10% N_2_/CO_2_ mixture is applied. Reactor feed consisted of the following components: arabinogalactan, pectin, xylan, potato starch, glucose, yeast extract, pepton, mucin, and cystein. The first two reactors operate on a fill-and-pull basis to simulate the different stages of food intake and digestion. Peristaltic pumps add a specific amount of feed and pancreatic, NaHCO_3_, and biliary fluids. The corresponding reactors are emptied at particular intervals. The last three compartments simulate the large intestine. These reactors with constant volume and under pH control are continuously mixed by shaking. The environmental conditions in each section of the system are entirely computer-controlled. This model also requires a stabilization phase for the intestinal microbiome. SHIME colonic compartment inoculation is performed with microbiota isolated from the faecal material of one individual to prevent artificial diversity. A typical SHIME experiment consists of four phases [31]:

- Two weeks stabilization period—to allow the microbial community to adapt to the environmental conditions in the respective colonic regions;
- Two weeks baseline period—in which the reactor is operated at nominal conditions and baseline parameters are measured;
- 2–4 weeks treatment period—during which the effect of a specific treatment on the gut microbial community is studied;
- Two weeks washout period—to determine how long the changes induced by the test substance can still be measured in the absence of the substance itself.

There are also modifications of the SHIME model. A TWINSHIME model can be used to test control and test samples simultaneously—two SHIME systems run in parallel, and all the environmental parameters are completely identical [32]. A modification of the model is also M-SHIME (Mucus-SHIME) [33]. In this model, the mucosal compartment is integrated with the colonic regions of SHIME, allowing the microbiota to adhere to the intestinal mucus layer under representative conditions. 

This model has the advantage of including all sections of the digestive tract. Computer control ensures controlled conditions. However, it does not take into account peristaltic movements and absorption of components. Particularly noteworthy is the M-SHIME modification, which allows the simulation of mucosal interactions.

**Figure 1:**
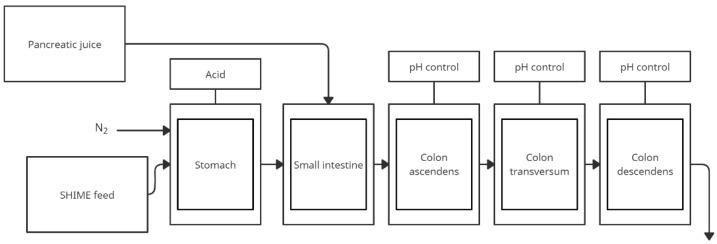
Simplified scheme of the SHIME model (adapted from de Wiele et al. [31]).

The figure below (Figure 1) shows a simplified scheme of the SHIME model. The model is often used to study the composition of the microbiome after specific nutrition as well as modification by plant substances, as shown in Table 1.

### 4.2. The SIMGI—SIMulator Gastro-Intestinal

**Figure 2:**
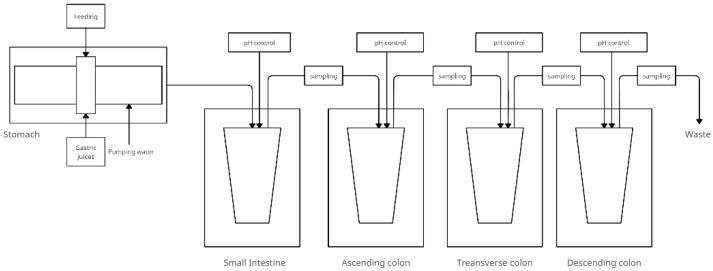
Simplified scheme of the SIMGI model (adapted from Barosso et al. [44]).

The SIMGI—a fully automated gastrointestinal multi-chamber simulator- is located in the Institute of Food Science Research in Spain. SIMGI aims to simulate the human gastrointestinal tract by evaluating gastrointestinal digestion processes and colonic fermentation of food and food ingredients A schematic of the model is shown in Figure 2. This system consists of compartments including the stomach, small intestine, and colon (ascending, transverse, and descending) with ports between the vessels for sampling [44]:

- The stomach—consists of two cylindrical transparent and stiff modules of methacrylate plastic covering a reservoir with flexible silicone walls. The gastric contents are mixed by peristaltic movements obtained by varying the water pressure flowing in the jacket between the plastic modules and the tank. The system allows the pH setting and emptying time to be changed into the small intestine. There are ports that allow nutrients, acid, or gastric juices to enter. The pH is controlled by a computer. The temperature of the gastric contents is maintained at 37 °C by pumping water;
- The small intestine consists of a double-walled, constantly magnetic stirred (at 150 rpm) glass reactor vessel that receives the gastric contents mixed with pancreatic juice and bile. Digestion time is 2 h at 37 °C and maintained at pH 6.8;
- The large intestine—fermentative module of the system. Stages of the large intestine are simulated in three anaerobic, double-walled glass reactors, and the contents of the colon are maintained at 37 °C. The pH is controlled by adding 0.5 M NaOH and 0.5 M HCl to maintain values of 5.6 ± 0.2, 6.3 ± 0.2, and 6.8 ± 0.2 in subsequent compartments.

Of particular relevance to the SIMGI model is the use of the gut microbiota. The model requires the development of a colonic-specific microbial community that must be stabilized before the start of experimental studies. This preliminary step allows the evolution of microorganisms in three reactors, from a faecal inoculum to a microbiota specific to the colonic region. The evolution of the gut microbiota in the ascending colon, transverse colon, and descending colon compartments of the SIMGI model was followed during a two-week stabilization period [45]. 

The advantage of the model is the presence of sections of the gastrointestinal tract preceding the large intestine, which gives a fuller representation of the transformations in one model. An additional advantage is simulated peristaltic movements of the stomach as opposed to the SHIME model. Figure 2 shows the simplified model and Table 2 shows the research conducted with the SIMGI model.

### 4.3. PolyFermS—Polyfermentor Intestinal Model

**Figure 3:**
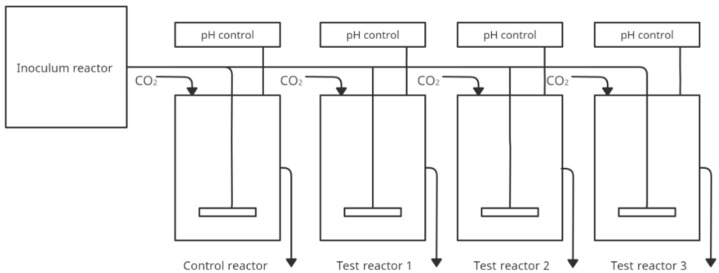
Simplified scheme of the PolyfermS model (adapted from Ziehler Berner et al. [52]).

In addition to models that simulate more sections of the gastrointestinal tract due to the important contribution of the large intestine and, more specifically, the gut microbiota to health effects, models have also been developed for this particular gastrointestinal section. For this purpose, a new Polyfermentor Intestinal Model (PolyFermS) was developed to compare the impact of different therapies on the same gut microbiota. PolyFermS consists of an inoculated with immobilized fecal microbiota. It continuously inoculates with the same microbiota different second-stage reactors assembled in parallel. The model uses fecal samples from healthy humans immobilized in diameter gel beads consisting of gellan gum, xanthan gum, and sodium citrate. The proposed conditions for faecal collection and immobilization of microbiota were described in Cleusix et al. [51]. Reactors containing 140 mL of nutrient medium have controlled pH and anaerobic conditions. Beads are colonized for 48 h in batch cultures under gut-like conditions (T = 37 °C; pH 5.7, control with 2.5 N NaOH, continuous flow of pure CO_2_ in the reactor airspace). Samples can be collected from each vessel. Figure 3 shows a simplified construction of the model. The table below (Table 3) shows publications in which the PolyFermS model was used.

### 4.4. The TIM-2 Gastro-Intestinal Model

One of the models used in simulating colonic conditions is the TIM-2 model described by Cuevas-Tena et al. [65]. This computer-controlled bioreactor-based system simulates peristaltic movement and absorption [66]. This system is based on the TIM-1 system (the TNO in vitro gastrointestinal model of the stomach and small intestine), which is successfully applied.

**Figure 4:**
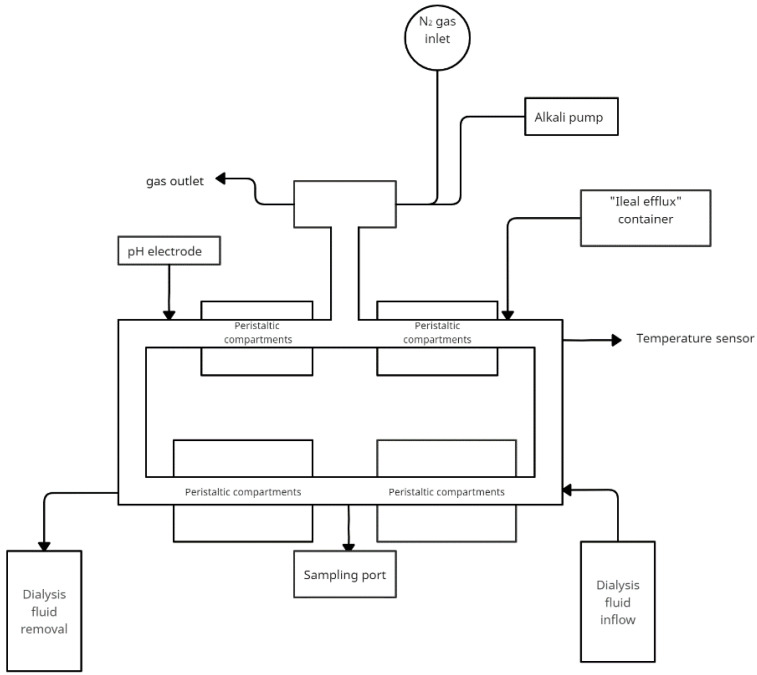
Simplified scheme of the TIM-2 model (adapted from Rehman et al. [67]).

The TIM-2 system is a dynamic, computer-controlled model that simulates the proximal portion of the human colon. The system’s main components are interconnected glass vessels with a flexible wall in the middle. Between the elastic and the glass wall water is pumped in at equal intervals—this simulates the peristaltic movements of the intestines. To maintain physiological conditions, water and fermentation products are removed through a dialysis system. Food is supplied through an inlet system. It is a simulated ileal effluent medium (SIEM). In composition, it mimics the components that reach the colon from the terminal ileum. The system has constant conditions of 37 °C, anaerobic conditions, and a constant pH = 5.8 controlled by a set of sensors. Figure 4 shows a simplified model of TIM-2. Model use human microbiota. A 16 h incubation period follows the introduction into the system. This is followed by a starvation period to consume all nutrients and the start of the test.

The TIM-2 system stands out from the rest because of its innovative solutions. The dialysis system allows better reproduction of in vivo conditions, as well as simulated peristaltic movements that better represent natural conditions than mixing. It is widely used in research on food, drugs and plant substances (Table 4). However, the study only includes the large intestine, to investigate the impact of previous sections TIM-1 must be used. The disadvantage of this solution is also the high cost.

### 4.5. Proximal Environmental Control System for Intestinal Microbiota (ECSIM)

**Figure 5:**
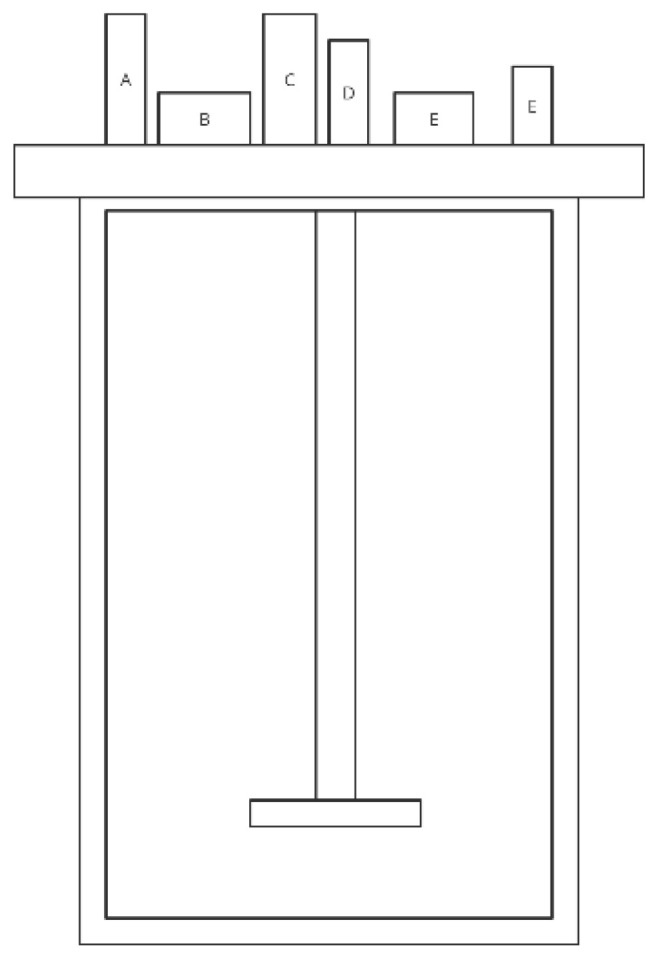
Simplified scheme of the ECSIM model (adapted from Brugère et al. [81]) The sensors A–F located in the steel plate represent temperature sensor, pH electrode, redox electrode, liquid or foam level sensor, injection input for pH correction and sterile sampling device.

ECSIM (Environmental Control System for Intestinal Microbiota) is a modular system consisting of three reactors and can be used in different configurations (independently or associated) to mimic different functions of the human colon, depending on which part it simulates f.e. P-ECSIM, T-ECSIM and D-ECSIM. It is created by Global Process Concept in France. Each bioreactor consists of a 2-L tank surrounded by a water jacket and a stainless steel top plate. The system includes a temperature sensor, a pH electrode, a redox electrode, a liquid or foam level sensor (modular), and an injection input for pH correction (which can be used for substrates), as well as a sampling device for inoculation, adding and taking sterile medium (Figure 5). The parameters are controlled by a computer programme. This medium is derived from those previously described and is a mixture of three solutions: a trace element solution, a vitamin solution (1 mL of each per 1 L of artificial gut medium) and a basal medium [79,80].

Modifications to ECSIM also include combining reactors to simulate all parts of the intestine—3S-ECSIM (three stage ECSIM). This study is, however, a long process because it requires stabilization at different stages, which can last up to 10 days [81]. The ECSIM model is not frequently used in scientific research. Table 5 presents a study conducted using this model.

### 4.6. EnteroMix

**Figure 6:**
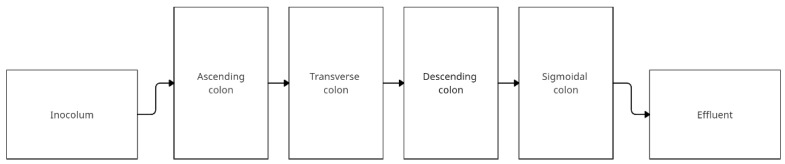
Simplified scheme of the EnteroMix model (adapted from Lamichhane et al. [84]).

The Enteromix system developed by Mäkivuokko et al. simulates four sections of the colon using four vessels representing the ascending, transverse, descending, and sigmoid colon. Before the system is placed a vessel with fresh medium, and after the system is placed a vessel for efluent. The distinguishing feature of this model is the seeding of small volumes—3, 5, 7, and 9 mL, respectively. The pH of the system is computer-controlled and is 5.5, 6.0, 6.5, and 7.0 for the individual colon sections. The system is not temperature controlled via water jackets and should be kept in a thermostatic room at 37 °C Anaerobic conditions are ensured by supplying N_2._ Flow occurs under gas pressure in pulses of 3 mL once every 3 h. First, the intercolum is incubated for 3 h, then pumped to ascending colon vessel, after 3 h to transverse colon vessel, and continuing to the following vessels. The whole experiment lasts 48 h. The final volumes of the vessels are 6, 8, 10, and 12 mL [83]. The scheme below (Figure 6) shows a simplified construction of the model. Table 6 shows the studies using Enteromix.

### 4.7. Summary of the Models

The table below (Table 7) summarizes the properties of each model.

The proposed models differ in their sophistication in simulating the digestive system and technological solutions. The choice of the digestive system area to simulate can be guided by the degree of model development. To simulate only one area such as ascending colon ECSIM model or PolyFermS where comparing with control group can by conducted. Adavantage of using ECSIM model is to use containers separately or in association. This allows researchers to conduct the experiment in different configurations, e.g., one inoculum with different conditions, one condition with another inoculum, three independent condition and intercolum or replicates of the same conditions. On the other hand, to simulate longer sections of the gastrointestinal tract from the stomach to the colon, the SHIME or SIMGI model would be a good option. SIMGI has the advantage of using a peristaltic pump, which can simulate the peristaltic movements of the stomach for mixing the ingested food with gastric fluids. The most advanced model with a peristaltic pump is TIM-2 model where the peristaltic passage of medium is used over the entire area. In this model is no differation in areas of colon. However, thanks to peristaltic movements, it is the most advanced. This model does not simulate gastrointestinal conditions because the TIM-1 model was created for this purpose. All of these models use relatively large medium volumes. Otherwise, the EnteroMix model uses a maximum of 12 mL. The disadvantage of this model, however, is the lack of temperature control through the water jacket, so that the ambient temperature must be at the right level. 

## 5. Possibility to Maintain a Healthy Gut Microbiome

There are many ways to protect normal gastrointestinal tract microbiota. These include the intake of probiotic, prebiotic, or synbiotic preparations that combine the two former ones [89]. Another method that can be used in parallel is using drugs containing ingredients that allow control of their release in the gastrointestinal tract, thus preventing excessive disturbances in intestinal microbiota composition [90].

### 5.1. Probiotics

Probiotics are defined by the World Health Organization (WHO) as living microorganisms that, if taken in certain amounts, can favorably affect the body. Probiotics can be delivered to the body along with food (dairy fermented drinks, vegetable or fruit silages) or in the form of dietary supplements [91]. Bacteria of the genus *Lactobacillus* and *Bifidobacterium* include species with described probiotic potential. Less frequently used (mainly in the animal context) include bacteria such as *Escherichia*, *Bacillus* and *Enterococcus*, as well as *Saccharomyces boulardii* yeast [92]. Probiotic microorganisms can regulate the immune system and stimulate the proliferation of intestinal epithelial cells, thus strengthening the intestinal barrier. Probiotics have the ability to restore the balance between microorganisms colonizing the intestines by reducing the number of pathogenic bacteria. Several mechanisms of probiotic strains action have been described in the literature. The first mechanism involves lowering the pH value in a given section of the gastrointestinal tract. Another mechanism involves the production of compounds with antimicrobial activity. The action of probiotics may also consist of competition for the place of adhesion or nutrients. Probiotic preparations can be administered in prophylaxis and therapy of many diseases which are caused by quantitative or qualitative disturbances to gastrointestinal microbiota. They are most often used as a supportive treatment in infectious gastroenteritis and other inflammatory conditions of the gastrointestinal tract, as well as in functional disorders such as irritable bowel syndrome [93]. Many studies proved that the use of probiotics has positive effects in the prevention of antibiotic-associated diarrhea [94]. 

However, it must be remembered that studies on the beneficial effects of probiotics have some limitations. Not many studies include metagenomic analysis, evaluating only selected strains of bacteria, which does not fully illustrate the research findings. Metagenomics enables a detailed study of the gut microbiome and the assessment of the effect of probiotic strains on the whole microbiome [95]. It should also be considered that the results of the study are influenced by the strains used, the route of administration or time of use.

### 5.2. Prebiotics

Another way to protect the proper microbiota from the negative effects of antibiotics is the use of prebiotics. Prebiotics can be used to relieve the symptoms of vaginal mycosis, stomach ulcers and intolerance to lactose and egzema [96,97,98]. Prebiotics can be used alone or in combination with probiotics. Preparations containing both probiotic microorganisms as well as prebiotics promoting their growth and activity are called synbiotics. Prebiotics are non-digestible nutrients whose task is to selectively stimulate the growth and/or activity of one or more strains of intestinal bacteria, resulting in a positive effect on the host’s health. Substances that may be referred to as prebiotics must meet many requirements. First of all, they cannot be affected by digestive enzymes, nor can they be absorbed in the upper parts of the digestive tract. Prebiotic substances are fermented in the large intestine. Bacteria colonizing the intestine use them as a source of energy and carbon for fermentation processes, resulting in short-chain fatty acids’ formation. Fermentation processes occurring in the intestine may result in a lowering of pH, increasing fecal mass, and reducing the amount of final nitrogen products and fecal enzymes [99]. 

Research shows the positive effect of prebiotic consumption on intestinal microbiota composition and metabolic activity. Advantages of prebiotic substances also include lowering LDL cholesterol, increased absorption of many elements, stimulation of the immune system, and regulation of pH prevailing in the intestines. Prebiotics reduce the amount of pathogenic intestinal bacteria also minimize the risk of bowel cancer [100]. The most frequently used prebiotic substances are fructans, in particular fructooligosaccharides and inulin. Their chemical structures consist of a chain of fructose units with terminal glucose unit linked by β-(2-1) glycosidic bond. Because human enzyme can digest only polysaccharides with α-glycosidic bonds fructooligosaccharides are indigestible and can reach the large intestine becoming a fuel for bacteria. Naturally, prebiotic substances are found in chicory, garlic, asparagus and artichokes [101].

### 5.3. Effect of Polyphenols on Microbiota 

In addition to fructooligosaccharides, substances of plant origin such as catechins, anthocyanins, and proanthocyanidins have prebiotic activity. Polyphenols are transformed by the digestive system and their undigested part reaches the second intestine where they are further broken down but by intestinal bacteria. A number of studies have assessed their effects on intestinal microbiome. The study also assessed the levels of short-chain fatty acids (SCFAs)—acetate, propionate, and butyrate, which are the main metabolites produced in the colon by bacterial fermentation of dietary fibers and resistant starch. They influence the regulation of epithelial barrier integrity, are the primary source of energy for colonocytes, influence the regulation of energy balance, have an immune function and regulation of inflammatory response, and what is important they shape the intestinal microbiome through antimicrobial activity and lowering of pH [13,102]. In animal studies, it was confirmed that anthocyanins from bilberry extract, anthocyanins and proanthocyanidins from arctic berry extracts, grape pomace extract, proanthocyanidin A from cinnamon bark extract, catechins and caffeine (green tea, black tea and oolong tea water extracts) have a growth-promoting effect on amount of SCFAs formed [103,104,105].

Many studies on animals confirm that anthocyanins, anthocyanidins and catechins stimulate the growth of health-promoting bacteria such as *Akkermansia, Lactobacillus* and *Bifidobacterium* and *Roseburia* [105,106,107,108] This has also been confirmed in clinical trials. Anthocyanins consumed with wild blueberry drink caused an increase in *Lactobacillus acidophilus* and Bifidobacterium after 6 weeks of application [109]. Consumption of 0.45 g or 1.8 g of pomegranate extract can reshape the gut microbiota, mainly through the modulation of Faecalibacterium, Odoribacter, and Parvimonas [110].

However, there is also an inverse relationship between the microbiome and polyphenols. The gut microbiome influences the bioavailability of polyphenols. Some people have been found to produce equol and O-desmethylangolensin from isoflavones which may be related to going through menopause as not every woman produces these metabolites because of present of specific bacteria [111]. An interesting example of this is the formation of urolithins from ellagic acid and ellagitannins which is associated with differences in the colon microbiota. In a number of preclinical studies, urolithins have been shown to protect against aging and age-related diseases of the muscles, brain, joints and other organs [112].

## 6. Conclusions and Future Perspective

Many models are used to study the effects of selected ingredients on the microbiome. The main problem of these studies is the lack of a uniform methodology. Individual models differ in many parameters such as pH, the volume of fluids, type of medium used, testing time, and even the complexity of the model construction. A scientific consensus on the application is needed. Interestingly, in view of this need for harmonization for simulated digestion, a protocol for simulated digestion conditions was developed by the international INFOGEST network and multidisciplinary experts from more than 35 countries.

In vitro models, despite their disadvantages, are an interesting perspective for the development of gastrointestinal research. Some models better simulate natural bowel conditions by taking into account peristatic movements or by applying a dialysis system to the model (TIM-2). However, this involves higher costs. Improved versions of already known models and their enrichment, e.g., artificial membranes such as in the M-SHIME model, is an interesting perspective for the development of methods.

## Figures and Tables

**Table 1:** Selected studies using the SHIME model.

Investigated Effect	Publication
The behavior of Bacillus coagulans Unique IS2 spores during passage through the simulator of human intestinal microbial ecosystem	Ahire et al. [34]
Predicting and testing bioavailability of magnesium supplements	Blancquaert et al. [35]
Effect of Bifidobacterium crudilactis and 3′-sialyllactose on the toddler microbiota	Bondue et al. [36]
Differences between human urolithin-metabotypes in gut microbiota composition, pomegranate polyphenol metabolism, and transport along the intestinal tract	García-Villalba et al. [32]
Bacillus subtilis HU58 and Bacillus coagulans SC208 probiotics reduced the effects of antibiotic-induced gut microbiome dysbiosis	Marzoratio et al. [37]
The ability of antioxidant vitamins and the prebiotics FOS and XOS to diversify the composition and function of the microbiota and improve the intestinal epithelial barrier may	Pham et al. [38]
Effects of human milk oligosaccharides on the adult gut microbiota and barrier function	Šuligoj et al. [39]
Prebiotic effects of carrot RG-I on the gut microbiota of four human adult donors	Van den Abbeele [40]
Evaluation of prebiotic properties of a commercial artichoke inflorescence extract revealed bifidogenic effects	Van den Abbeele et al. [41]
Modulation of the microbial community by aronia (*Aronia melanocarpa*) polyphenols	Wu et al. [42]
Interindividual variability of soil arsenic metabolism by human gut microbiota	Yin et al. [43]

**Table 2:** Selected studies using the SIMGI model.

Investigated Effect	Publication
The behavior of citrus pectin during digestion and its potential prebiotic properties	Ferreira-Lazarte, Alvaro et al. [46]
The effect of chia seed mucilage on the bioaccessibility of glucose, dietary lipids and cholesterol along the gastrointestinal tract.	Tamargo, Alba et al. [47]
Modifications and potential effects of AgNPs with food applications during their passage through the digestive tract	Cueva, Carolina et al. [48]
Metabolic activity of probiotics at the intestinal level, and in particular, to assess the impact of probiotic supplementation in the microbial metabolism of grape polyphenols.	Gil-Sánchez, Irene et al. [49]
Impact of red wine on colonic metabolism	Cueva, Carolina et al. [50]

**Table 3:** Selected studies using the Polyferm S model.

Investigated Effect	Publication
Modeling of chicken cecal microbiota ecology and metabolism	Asare et al. [53]
Effect of storage on planktonic and sessile artificial colonic microbiota	Bircher [54]
Effect of dietary nucleosides and yeast extracts on composition and metabolic activity of infant gut microbiota	Doo et al. [55]
Effect of iron on butyrate production by the child’s gut microbiota in vitro	Dostal et al. [56]
Clostridium difficile colonization and antibiotics response in elderly intestinal fermentation	Fehlbaum et al. [57]
Modulatory effects of Lactobacillus paracasei CNCM I-1518 on composition and function of elderly gut microbiota	Fehlbaum et al. [58]
Bistable auto-aggregation phenotype in Lactiplantibacillus plantarum	Isenring [59]
In Vitro Gut Modeling as a Tool for Adaptive Evolutionary Engineering of *Lactiplantibacillus plantarum*	Isenring et al. [60]
Inhibitory Activity of Microcin J25 (bacteriocin produced by *Escherichia coli)* Against Salmonella Newport	Naimi et al. [61]
Modulation of lactate metabolism by faecal inoculum, pH and retention	Pham et al. [62]
Prebiotic potential of different dietary fibers	Poeker et al. [63]
Synergistic effects of Bifidobacterium thermophilum RBL67 and selected prebiotics on inhibition of Salmonella colonization	Tanner et al. [64]

**Table 4:** Selected studies using the TIM-2 model.

Investigated Effect	Publication
Prebiotic Effect of Lactulose	Bothe et al. [68]
Effect of potato fiber on survival of Lactobacillus species at simulated gastric conditions and composition of the gut microbiota	Larsen et al. [69]
Effects of functional pasta ingredients on different gut microbiota	Martina et al. [70]
Prebiotic effects of pectooligosaccharides obtained from lemon peel	Míguez et al. [71]
Potential of high- and low-acetylated galactoglucomannooligosaccharides as modulators of the microbiota composition	Míguez et al. [72]
Investigation of changes in gut microbiota upon feeding predigested Hibiscus sabdariffa, Agave fructans and oligofructans (OF)	Sáyago-Ayerdi et al. [73]
Bioconversion of polyphenols and organic acids by gut microbiota of predigested Hibiscus sabdariffa L. calyces and Agave (A. tequilana Weber) fructans	Sáyago-Ayerdi et al. [74]
Bioconversion by gut microbiota of predigested mango (Mangifera indica L) ‘Ataulfo’ peel polyphenols	Sáyago-Ayerdi et al. [75]
Prebiotic effect of predigested mango peel	Sáyago-Ayerdi et al. [75]
Modulation of the microbiome by citrus fruit extract	Sost et al. [76]
Eeffect of a blend of three mushrooms (Ganoderma lucidum GL AM P-38, Grifola frondosa GF AM P36 and Pleurotus ostreatus PO AM-GP37)) on gut microbiota composition	Verhoeven et al. [77]
Impact of a fermented soy beverage supplemented with acerola by-product on the gut microbiota	Vieira et al. [78]

**Table 5:** Selected studies using the ECSIM model.

Investigated Effect	Publication
Evaluation of the viability and resuscitability of microorganisms after preservation with certain cryoprotective agents (CPAs)	Tottey et al. [82]

**Table 6:** Selected studies using the EnteroMix model.

Investigated Effect	Publication
Effects of lactose on colon microbial community structure and function	Mäkivuokko et al. [83]
The efect of 2′-fucosyllactose on simulated infant gut microbiome and metabolites	Salli et al. [85]
In vitro effects on polydextrose by colonic bacteria and caco-2 cell cyclooxygenase gene expression	Mäkivuokko et al. [86]
The effects of polydextrose and xylitol on microbial community and activity in a 4-stage colon simulator	Mäkivuokko et al. [87]
Synbiotic effects of lactitol and Lactobacillus acidophilus NCFM™	Mäkivuokko et al. [88]

**Table 7:** Summary of the Models.

	Simulated Areas of the Digestive System	Volume	Control of Temperature	Control of Anaerobic Condition	Modification Options	Simulating Peristaltic Movements
**SHIME**	Stomach, small intestine, acending colon, transverse colon, descending colon	500 mL	Water jacket	flow of N_2_ gas or 90/10% N_2_/CO_2_	M-SHIME TWINSHIME	
**SIMGI**	Stomach, small intestine, acending colon, transverse colon, descending colon	250, 400 and 300 mL (for specific compartments)	Water jackets	flow of N_2_ gas		Peristaltic movement in simulated stomach
**PolyFermS**	Colon (no differentiation)	200 mL	Water jackets	flow of CO_2_		
**TIM-2**	Colon (no differentiation)	120 mL	Water jackets	flow of N_2_ gas		Peristaltic movements along the entire length of the model
**ECSIM**	Small intestine, acending colon, transverse colon, descending colon used separately or combined	1000 mL	Water jackets	N_2_ flush, and then maintained by the fermentative activity of the microbiota.	P(roximal)-ECSIM T(ransversal)-ECIM D(escending)-ECSIM 3S-ECSIM (all with slow or normal transit)	
**EnteroMix**	Ascending colon, transverse colon, descending colon, sigmoidal colon	6, 8, 10, 12 mL (for specific compartments)	Ambient temperature control	N_2_ flush		

## Data Availability

Not applicable.
